# A Rare Case of Spontaneous Isolated Dissection of the Superior Mesenteric Artery

**DOI:** 10.7759/cureus.4725

**Published:** 2019-05-23

**Authors:** Samia Asif, Aref Qureini, Joseph Bennett

**Affiliations:** 1 Internal Medicine, University of Missouri Kansas City (UMKC), Kansas City, USA

**Keywords:** superior mesenteric artery dissection, atrial fibrillation with rapid ventricular rate

## Abstract

Isolated spontaneous dissection of the superior mesenteric artery (SMA) is a rare entity that is increasingly becoming recognized due to an improvement in imaging techniques. The pathogenesis of a spontaneous SMA dissection has yet to be fully elucidated. Here, we present the case of isolated SMA dissection in a 65-year-old female who was seen in the emergency room with acute substernal chest and left upper quadrant abdominal pain. She was managed for atrial fibrillation with a rapid ventricular response. She underwent computed tomography (CT) angiogram of the chest, abdomen, and pelvis, which revealed focal dissection involving SMA measuring 2.7 cm in width. Vascular surgery recommended conservative management with low-dose daily aspirin and the optimization of blood pressure control. She subsequently was seen as an outpatient with complete resolution of abdominal pain. Given the low incidence rate, vascular surgery evaluation may be required to determine the best course of management. Treatment needs to be individualized for each patient. Since abdominal pain is a common complaint for which patients are seen in each clinical setting, it is important to highlight this case to create awareness regarding the possibility of isolated SMA dissection as one of the underlying etiologies.

## Introduction

Isolated spontaneous dissection of the superior mesenteric artery (SMA) is a rare entity that is increasingly becoming recognized due to an improvement in imaging techniques. Due to its infrequent occurrence, clinical experience regarding the management of isolated SMA dissection is primarily based on outcomes from reported cases in the literature. Given its presentation as acute or chronic abdominal pain, a common but nonspecific complaint encountered in routine practice, it remains essential that all medical professionals be aware of SMA dissection as a possible underlying etiology. Here, we present the case of a patient who presented with chest and abdominal pain and was found to have an isolated SMA dissection.

## Case presentation

A 65-year-old Caucasian lady with a past medical history of essential hypertension and poorly controlled type 2 diabetes mellitus was seen in the emergency room (ER) with acute onset retrosternal chest and left upper abdominal pain that woke her up from sleep. She described the abdominal pain as sharp, non-radiating, and worsening with respiration. The chest pain was reported as pressure-like in quality. She endorsed nausea, vomiting, and dyspnea. In the ER, her pulse rate was noted to be irregular and high at 155 beats per minute (bpm) and her blood pressure was noted to be 149/108 mmHg.

**Figure 1:**
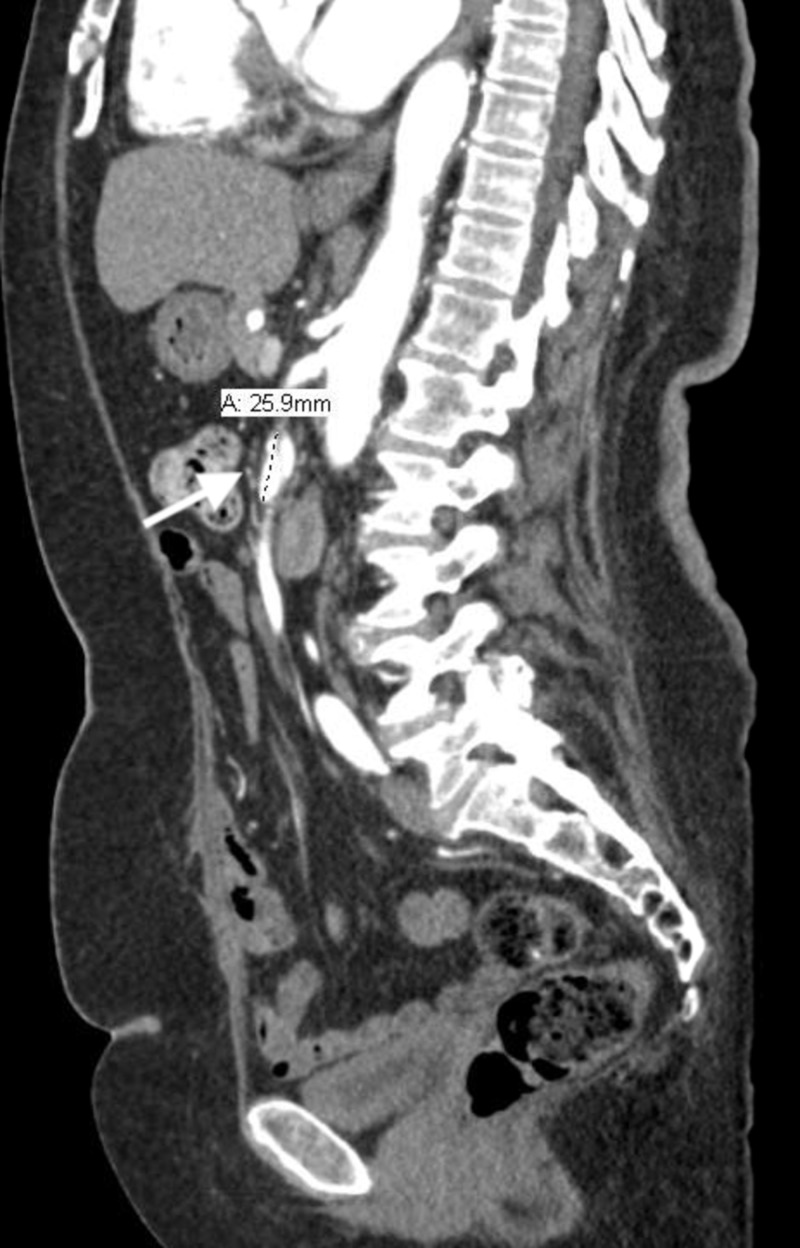
Sagittal view of isolated superior mesenteric artery dissection (indicated by arrow) on a CT angiogram.

**Figure 2:**
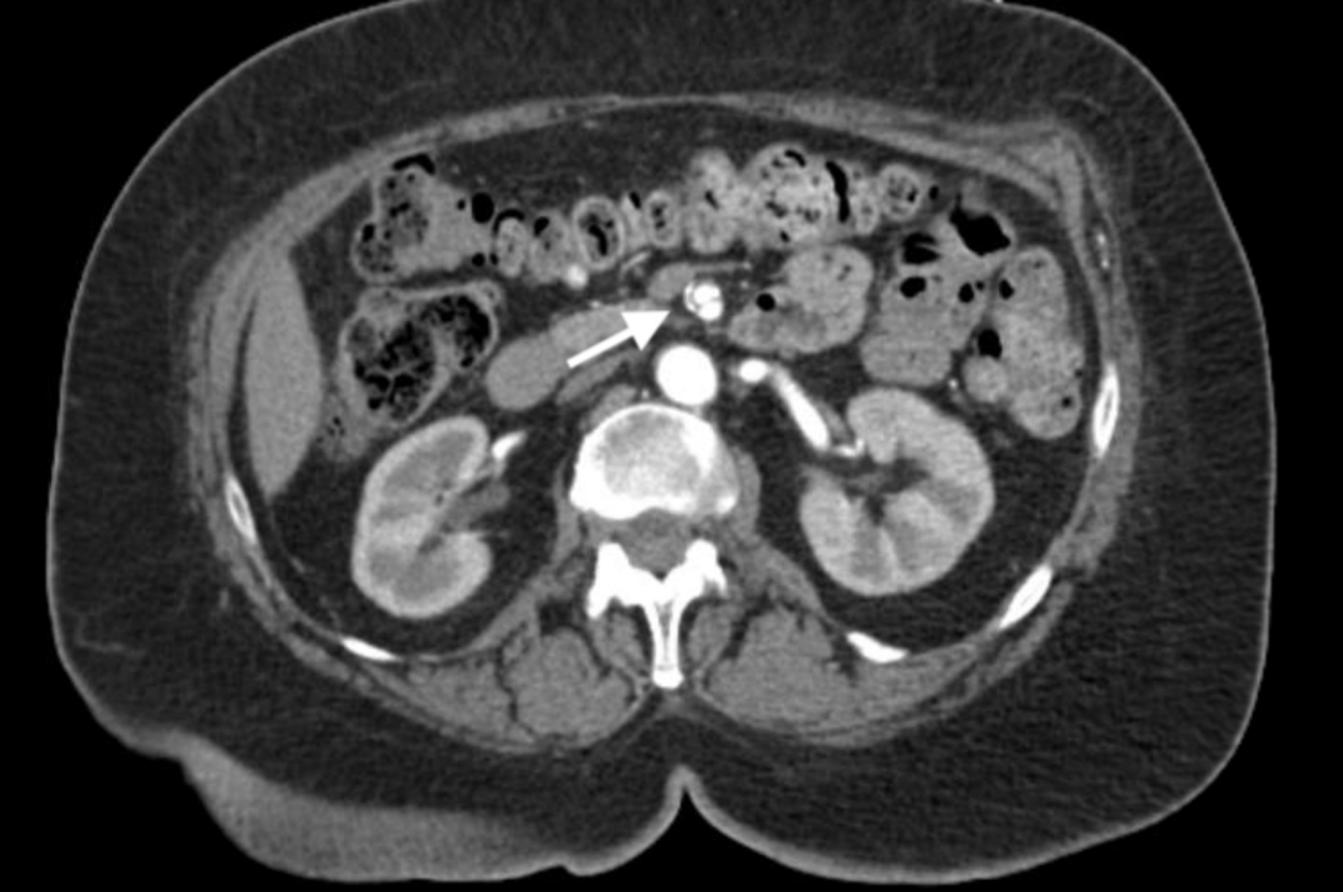
Cross-sectional view of isolated superior mesenteric artery dissection (indicated by arrow) on a CT angiogram.

Initial workup showed normal electrolytes and renal function tests as well as elevated blood sugar levels at 392 mg/dL. Lactic acid levels were normal at 1.7 mmol/liter. Hemoglobin A1c was 11.6%. The electrocardiogram (EKG) showed atrial fibrillation (AF) with rapid ventricular response (RVR). A chest X-ray was done, which showed mediastinal widening. This was followed by a computed tomography (CT) angiogram of the chest, abdomen, and pelvis, which revealed a focal dissection involving the superior mesenteric artery (SMA) measuring 2.7 centimeters (cm) in width but, otherwise, the normal caliber of the aorta without dissection or intramural hematoma was noted (Figures 1-2).

She was on three different antihypertensive agents and reported her hypertension was not optimally controlled and her medications were being adjusted as an outpatient by her primary care provider. Intravenous esmolol infusion to minimize arterial wall shear stress and for rate control of AF with RVR was initiated; this was followed by resolution of the chest pain. She was later transitioned to oral metoprolol. Troponin levels were trended and remained negative.

Vascular surgery was consulted and recommended conservative management with daily low-dose aspirin and the optimization of blood pressure control. A repeat CT angiogram of the abdomen and pelvis was done three days later due to continued complaints of left upper quadrant pain; this showed unchanged focal dissection measuring 2.6 cm in length and causing less than 50% stenosis of the true lumen was noted. This was reviewed by vascular surgery who continued to recommend medical management. Her risk for thrombotic stroke in the setting of AF calculated using the CHA2DS2-VASc scoring criteria (which includes a history of congestive heart failure, hypertension, age more than 75 years, diabetes mellitus, prior stroke or transient ischemic attack, vascular disease history, age more than 65 years, and gender) was three. Accordingly, anticoagulation was initiated with rivaroxaban. She was discharged home in stable condition on the fifth day of admission and at the two-week outpatient follow-up, she reported complete resolution of abdominal pain.

## Discussion

While aortic dissection can extend into branching arteries, spontaneous and isolated dissection of a visceral artery, such as the superior mesenteric artery (SMA) is rare. Based on previously reported cases, isolated dissections have involved the renal arteries, coronary arteries, intracranial arteries, and then the superior mesenteric artery in order of frequency [1]. Among the gastrointestinal arterial supply, it is the most frequent digestive artery dissection to be reported [2]. The pathogenesis of a spontaneous SMA dissection has yet to be fully elucidated; in the past, associations have been noted with trauma, hypertension, atherosclerosis, cystic medial necrosis, and elastic tissue diseases (Marfan’s disease, Ehlers-Danlos disease) and fibromuscular dysplasia [3]. However, in most cases, no cause is found. In a study by Kimura et al., isolated SMA spontaneous dissection was more common in men as compared to females and hypertension was the most significant comorbidity [4].

Clinically, manifestations can vary and may be nonspecific; patients may have an acute presentation with abdominal pain, nausea, and vomiting or may report chronic abdominal pain with poor appetite and, possibly, weight loss. Occasionally, patients may be asymptomatic; rarely, patients may present with hemorrhagic shock resulting from rupture of the dissecting artery [5]. Given the improved sensitivity of CT imaging, more patients with asymptomatic SMA dissection are being identified [3]. Critical complications resulting from an isolated SMA dissection have been reported in the literature, including peritonitis, bowel ischemia, bowel necrosis, bowel rupture, and the development of aneurysm with subsequent rupture [4].

A CT angiography is a good initial diagnostic test, as it allows the visualization of a false lumen as well as the intimal flap. It also provides the dimensions of the lesion and may identify signs of acute bowel ischemia-like enhancement of the bowel wall or mesenteric edema [6]. Diagnosis by ultrasound imaging or magnetic resonance imaging (MRI) is possible as well [3]. However, catheter angiography remains the gold standard diagnostic test, though this should only be used if surgical or endovascular treatment is needed or in patients with worsening symptoms.

Treatment modalities include either conservative management or invasive management. Conservative management comprises anti-platelet therapy, optimizing blood pressure control, anticoagulation, and bowel rest. Invasive interventions include endovascular or surgical repair. In case of a deteriorating clinical condition, such as a lack of improvement in abdominal pain within one to two weeks or the development of complications such as bowel necrosis or aneurysms requiring repair, further invasive treatment strategies can be pursued [7].

Treatment needs to be individualized for each patient, taking into consideration the patient’s symptoms as well as the anatomy of the dissection, particularly the level of reduction in the diameter of the true lumen [3]. Given the rarity of occurrence, obtaining specialty evaluation from vascular surgery is important.

## Conclusions

Isolated spontaneous dissection of the SMA in the absence of aortic dissection is rare and unsuspected. SMA dissection as a cause of abdominal pain may remain unrecognized if the examining physician does not have a clinical suspicion for this and appropriate imaging studies are not ordered. This makes it essential to highlight this case as a potentially life-threatening cause of acute or chronic abdominal pain. Timely and accurate diagnosis allows correct treatment that is lifesaving.
